# Acute ischemic stroke after right middle lobectomy with favorable clinical outcome of mechanical thrombectomy: A case report

**DOI:** 10.1097/MD.0000000000043007

**Published:** 2025-07-04

**Authors:** Zijun Ren, Linzhuo Qu, Enbo Zhu, Guanglin Jin, Guanghui Dong, Guanglin Liu, Chunhua Quan, Hongjian Guan

**Affiliations:** a Department of Neurology, Stroke Center, Yanbian University Hospital, Yanji, Jilin, China; b Medical College, Yanbian University, Yanji, Jilin, China.

**Keywords:** acute ischemic stroke, case report, mechanical thrombectomy, pulmonary vein stump, right middle lobectomy

## Abstract

**Rationale:**

Acute ischemic stroke after lung resection is a rare and serious complication, often caused by a venous stump thrombus. This study presents the first reported case of cerebral embolism caused by a venous stump thrombus after right middle lobe resection and aims to raise clinical awareness and provide evidence for stroke prevention and management strategies in the perioperative period.

**Patient concerns:**

A 41-year-old woman who had undergone thoracoscopic right middle lobectomy for lung adenocarcinoma with right hemiplegia and complete motor aphasia was admitted to our hospital.

**Diagnoses:**

After obtaining clinical manifestations, and medical history, the final diagnosis was a cerebral embolism presumably caused by a thrombus in the pulmonary vein stump after right middle lobectomy for lung cancer.

**Interventions:**

An urgent mechanical thrombectomy was performed under general anesthesia. Following surgical intervention, subcutaneous injections of low molecular weight heparin calcium were initiated at a dosage of twice daily. This regimen was maintained for a period of 5 days.

**Outcomes:**

On postoperative day 6, the patient was transferred to the rehabilitation department with a modified Rankin Scale score of 2. Imaging studies revealed extensive infarction in the middle cerebral artery territory, along with intracerebral hemorrhage. The patient showed significant neurological improvement over time.

**Lessons:**

We reported a case of a patient who underwent mechanical thrombectomy for acute ischemic stroke after lobectomy for lung cancer. Although all test results were negative, the thrombus source in the patient was still considered to be due to thrombus formation at the residual pulmonary vein. Therefore, it is very important to further explore the etiological prevention and treatment strategies for the perioperative period.

## 1. Introduction

Perioperative acute ischemic stroke (AIS) is a relatively rare (approximately 0.868%),^[1]^ but devastating complication of lung resection.^[2]^ We largely believe that the free embolus causing neurological deficits in patients originates from the pulmonary vein stump, and emergency arterial thrombectomy stands as the optimal treatment for strokes induced by pulmonary vein thrombosis. Despite several reported cases of cerebral embolism linked to lobectomy, to our knowledge, cerebral embolism resulting from venous stump thrombosis post-right middle lobe resection remains unreported. We present a case of a middle-aged female patient who experienced a stroke the day after undergoing resection of the middle lobe of her right lung. Following a successful emergency endovascular mechanical thrombectomy, the patient showed positive outcomes.

## 2. Case report

The patient was a 41-year-old woman. A 0.6 × 0.5 cm ground-glass opacity lesion had been identified at the right middle lobe during incidental computed tomography (CT) screening 3 months ago when she was taking her physical examination. Results of all preoperative laboratory tests including electrocardiogram, blood examination and urine examination were within normal limits. She had no history or ongoing (chronic) health problems like hypertension, diabetes mellitus, cerebrovascular disease, and atrial fibrillation. She underwent right middle lung via video-assisted thoracic surgery for lung adenocarcinoma. After successful endobronchial anesthesia and nerve block anesthesia, the patient was placed in the left lateral decubitus position, and routine disinfection and draping were performed. The right middle pulmonary vein, the right middle pulmonary artery and its branches, as well as the right middle pulmonary trachea, were isolated. Subsequently, the middle lobe of the right lung was completely excised using a cutting and suturing device (canwell). Following the operation, an ultra-fine closed thoracic drainage tube was placed at the 8th intercostal space along the posterior axillary line, and the chest was closed layer by layer. After the procedure, the patient was extubated upon waking up and safely returned to the ward.

**Figure 1.**
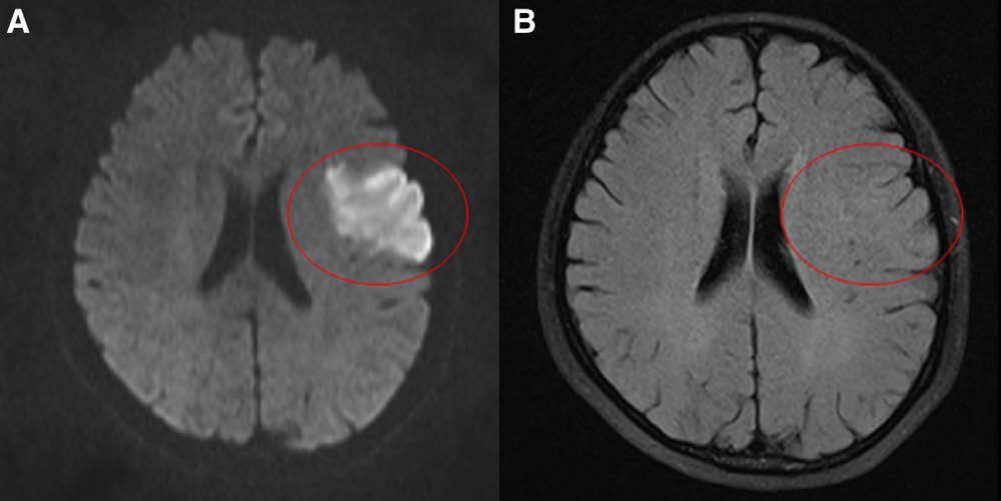
DWI showed high signal (A). FLAIR showed no obvious abnormalities (B). DWI = diffusion-weighted imaging, FLAIR = fluid-attenuated inversion recovery.

But the patient was transferred to neurology department for sudden complete motor aphasia and right complete hemiplegia the next morning after the surgery. Magnetic resonance imaging taken immediate after onset. Further examination via magnetic resonance imaging showed increased signal intensity in the left middle cerebral artery (MCA) territory on diffusion-weighted imaging (Fig. 1A). That was not seen on fluid-attenuated inversion recovery imaging (Fig. 1B). A diagnosis of left MCA occlusion with diffusion-weighted imaging/fluid-attenuated inversion recovery mismatch was made after detecting the abnormality. The National Institutes of Health Stroke Scale score was 11. Due to the patient presented with AIS from the day after surgery, so recombinant tissue plasminogen activator was not applicable.

**Figure 2.**
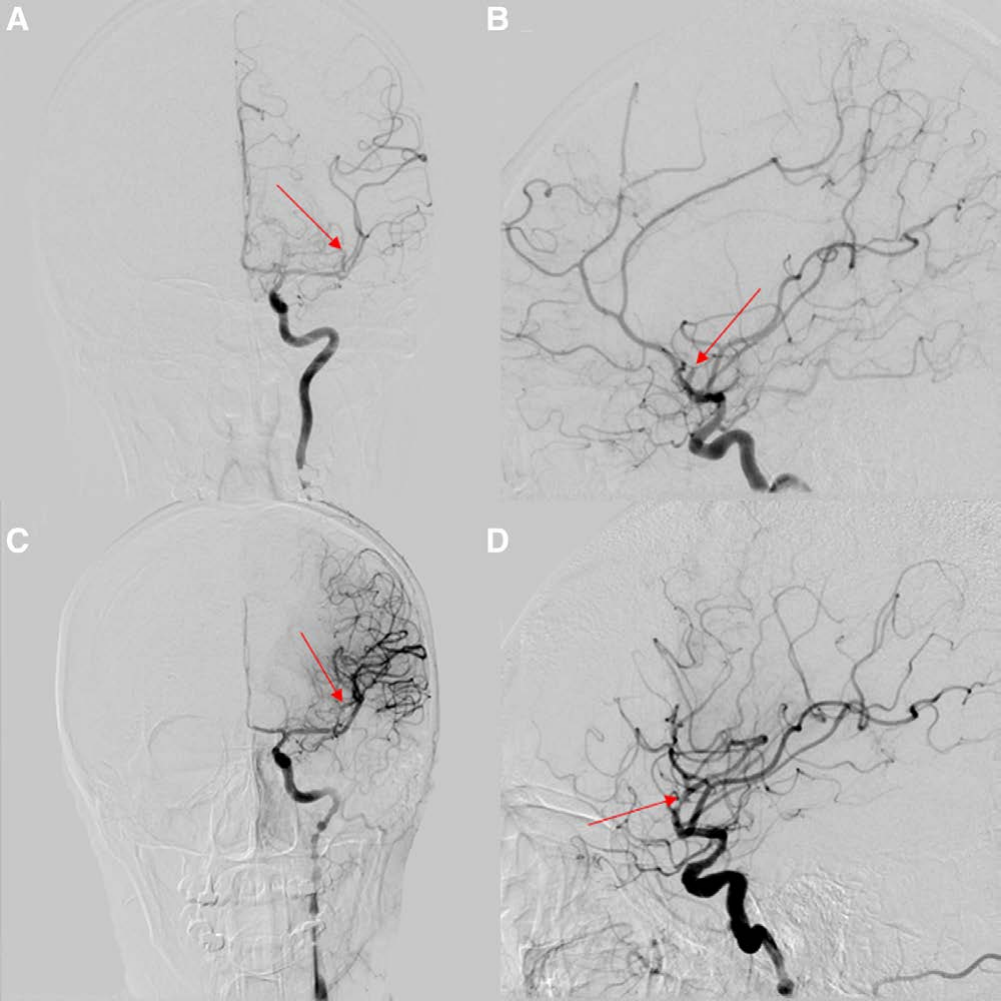
DSA revealed the left M2-MCA occlusion in front position (A). DSA showed the left middle cerebral artery was recanalization in lateral position (B). DSA revealed the left M2-MCA occlusion in front position (C). DSA showed the left middlecerebral artery was recanalization in lateral position (D). DSA = digital subtraction angiography, MCA = middle cerebral artery.

After a fast preoperative evaluation, an urgent mechanical thrombectomy was performed under general anesthesia. The patient was in a supine position. The right femoral artery serves as the puncture point. Neuro-interventionists kept an 8-French long sheath in her right femoral artery by seldinger technique after punctured successfully (08:34). Digital subtraction angiography revealed the left M2-MCA occlusion (Fig. 2A and B). An 8-French guiding catheter was navigated to the left internal carotid artery via the femoral approach along Terumo guide wire and multifunctional catheter. The Terumo guide wire and multifunctional catheter was withdrew. Then the Stryker intracranial support catheter was guided to the cavernous sinus segment of the left internal carotid artery along the Terumo guidewire, and sent the microcatheter to the left M2-MCA through the occlusion along the microcatheter. Then the SFR 4.0 × 20 mm stent was carefully sent to the distance of the occlusion under fluoroscopy, then released the stent for 5 minutes. The thrombus was pulled while aspirating the Stryker intracranial support catheter, we can saw the mass thrombus in the 50 mL syringe. Next, we reviewed the digital subtraction angiography and saw the left middle cerebral artery was recanalization (09:09) (Fig. 2C and D). The thrombolysis in cerebral infarction grade was 2B or above. The total time from femoral access to recanalization was 35 minutes and then the treatment was terminated.

**Figure 3.**
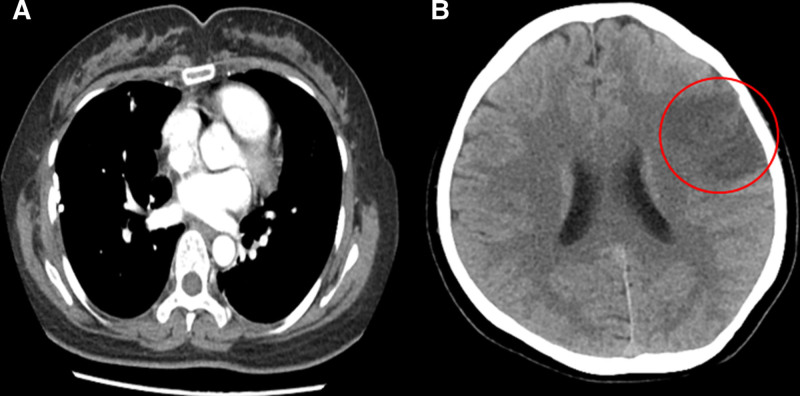
The contrast-enhanced CT scan conducted 3 days postoperatively revealed no thrombus in the pulmonary vein stump (PVS) (A). CT scan illustrated an extensive infarction in the MCA territory, accompanied by intracerebral hemorrhage (B). CT = computed tomography, MCA = middle cerebral artery.

The postoperative CT imaging revealed a significant hypodense region in the left cerebral hemisphere. Subsequently, a contrast-enhanced CT scan conducted 3 days postoperatively revealed no thrombus in the pulmonary vein stump (PVS) (Fig. 3A). We think the thrombus has already fallen off. Consequently, we commenced subcutaneous injections of low molecular weight heparin calcium twice daily. The subsequent CT scan illustrated an extensive infarction in the MCA territory, accompanied by intracerebral hemorrhage (Fig. 3B). Due to the effective medical management of the hemorrhage and associated swelling, surgical intervention for decompression was considered unnecessary.

The patient’s mental status and motor function showed gradual improvement over time. During this period, we conducted several physiological examinations, including Holter electrocardiography, venous ultrasonography, and carotid artery ultrasonography. Additionally, we performed blood tests to evaluate coagulation function and to detect the presence of autoantibodies associated with collagen diseases. However, no anatomical abnormalities or thrombophilia factors were identified. The patient was transitioned to a department of rehabilitation medicine on day 6 post-embolization, with a modified Rankin Scale score of 2.

## 3. Discussion

This report examines a rare case of cerebral embolism originating from the right middle lobectomy. Through literature review, we identified a total of 3 reported instances of cerebral infarction following right lung lobectomy.^[3,4]^ Adding this case, the total amounts to 4 cases (Table 1). The focus is on 2 critical aspects: the management of cerebral embolism associated with PVS and the application of mechanical thrombectomy in the treatment of stroke caused by acute large vessel occlusion. But after reviewing the literature, we found that the etiology of stroke caused by lobectomy is still unclear. Therefore, we should prevent and treat stroke complications from the perioperative period.

**Table 1:** Summary of clinical characteristics of the case series.

Case	Age	Sex	Preoperative complication	Location of lobectomy	Occluded sites	NIHSS score	Time from onset to puncture (min)	Time from puncture to recanalization (min)	Postoperative AF	mRS at 3 months
1^[3]^	48	F	None	RLL	Left MCA	12	122	150	None	1
2^[3]^	68	M	None	RUL	Left MCA	10	120	160	None	1
3^[4]^	–	–	None	RUL	Left MCA	19	103	122	None	0
4	41	F	None	RML	Left MCA	11	94	129	None	1

MCA = middle cerebral artery.

Thoracic surgeons should evaluate whether there is stenosis of cardiac and cerebral blood vessels and determine whether there is a risk of atrial fibrillation embolus shedding before lobectomy. Studies have shown that the incidence of pulmonary vein stump thrombosis is directly proportional to the increase in LA volume and CHA2DS2-VASc score, which can be used as a means of preoperative assessment^.[5]^ After preoperative assessment, targeted preventive medication can be given, and signs of postoperative complications can be detected in a timely manner. However, research findings indicate that patients without a prior history of vascular disease who are administered anticoagulants or antiplatelet drugs exhibit an elevated risk of cerebral infarction following lung resection.^[6]^ Therefore, whether preoperative prophylactic medication is effective requires further exploration. Other studies suggest that the risk of thrombosis is associated with the length of the pulmonary vein stump during surgery. Specifically, a longer residual length is correlated with an increased risk of thrombosis.^[7]^ Therefore, large sample experiments should be carried out to study the appropriate position for ligation when different lobes are excised. It is essential to leave an adequate length of the pulmonary vein stump, as this practice not only reduces the risk of thrombosis in the pulmonary vein stump but also minimizes the occurrence of complications such as bleeding and cardiac tamponade.^[8]^ As the cause of pulmonary vein thrombosis, it cannot be ruled out that intraoperative vascular endothelial injury leads to the accumulation of perioperative inflammatory factors affecting the hypercoagulable state.^[9]^ Another undisputed reason is the turbulent blood flow in the pulmonary vein stump after left upper lobectomy, which increases the probability of thrombosis from 13.5% to 17.9%, which is much higher than other types of lobectomy.^[10]^

Retrospective studies have shown that about 30% of poststroke patients did not find pulmonary vein stump thrombus in contrast-enhanced chest CT examination, and 50% of patients found thrombus in the left upper pulmonary vein stump. Although the mechanism is unclear, it is clear that lobectomy is an independent risk factor for stroke.^[11]^ Because no definite cause of embolus was found in this case, we suspected that it was caused by thrombus shedding from the stump of pulmonary vein. In this case, we commenced subcutaneous injections of low molecular weight heparin calcium twice daily. After continuous injection for 5 days, the patient was in stable condition and discharged on her own. In contrast, since most cases of ischemic stroke after lobectomy occurred during the postoperative acute phase, the initiation of anticoagulant therapy at an early stage after lobectomy is controversial due to the risk of hemorrhagic complications such as hemothorax.^[4]^ Literature data show that the incidence of cerebral infarction is high from the first day to the seventh year after surgery,^[10]^ and the incidence of postoperative atrial fibrillation does not exceed 30%.^[12]^ However, atrial fibrillation is the most common postoperative complication, and anticoagulation is the most important countermeasure. Nevertheless, long-term use of anticoagulants can lead to bleeding and abnormal coagulation rates, so further clinical trials are needed to determine whether use should be discontinued. Therefore, more data regarding the timing of administration and duration of anticoagulant therapy after ischemic stroke caused by lobectomy need to be accumulated in the future.

To detect pulmonary vein stump thrombus in advance, it is recommended to undergo at least 1 chest enhanced CT scan after surgery. Transesophageal echocardiography is also a useful examination for identifying pulmonary vein stump thrombus. However, due to the invasive nature of transesophageal echocardiography, it is advisable to regularly perform chest enhanced CT scans. If stump thrombus is detected, anticoagulation therapy should be initiated immediately.^[7]^ The most important thing after surgery is to prevent cancer recurrence, because cancer thrombus has the risk of stroke.

Finally, this study has several limitations. Firstly, it is based on a single case, larger, multi-center studies are necessary to validate the results. Additionally, the optimal timing and role of anticoagulation therapy post-surgery remain uncertain, highlighting the need for further clinical trials to establish standardized treatment protocols.

## 4. Conclusions

This case report presents the first documented instance of cerebral embolism caused by thrombus formation in the PVS following right middle lobectomy for lung cancer. The patient underwent successful mechanical thrombectomy, which led to significant neurological recovery, underscoring the importance of early diagnosis and timely intervention for large vessel occlusion. This case contributes to the understanding of stroke as a potential complication following lobectomy and highlights the effectiveness of endovascular treatment in improving outcomes for patients with AIS caused by thrombus shedding from the pulmonary vein stump. However, further research is needed to explore optimal perioperative management strategies, including preventive measures for thrombus formation and the ideal use of anticoagulation therapy. Larger, multi-center studies are crucial for refining treatment protocols, improving early detection, and enhancing recovery in patients undergoing lung resection, ultimately preventing similar complications in the future.

## Author contributions

**Conceptualization:** Zijun Ren, Linzhuo Qu, Hongjian Guan.

**Data curation:** Hongjian Guan.

**Formal analysis:** Zijun Ren, Enbo Zhu.

**Funding acquisition:** Hongjian Guan.

**Investigation:** Zijun Ren, Linzhuo Qu.

**Methodology:** Zijun Ren, Linzhuo Qu, Hongjian Guan.

**Project administration:** Hongjian Guan.

**Software:** Enbo Zhu.

**Supervision:** Guanglin Jin.

**Visualization:** Guanghui Dong, Guanglin Liu.

**Writing – original draft:** Zijun Ren, Linzhuo Qu.

**Writing – review & editing:** Chunhua Quan, Hongjian Guan.
